# Role of Neutrophils in Systemic Vasculitides

**DOI:** 10.3389/fimmu.2020.619705

**Published:** 2020-12-17

**Authors:** Despina Michailidou, Tomas Mustelin, Christian Lood

**Affiliations:** Division of Rheumatology, University of Washington, Seattle, WA, United States

**Keywords:** neutrophil extracellular traps, anti-neutrophil cytoplasmic antibody associated vasculitis, polyarteritis nodosa, Takayasu’s arteritis, giant cell arteritis, Behcet’s disease

## Abstract

Neutrophils and neutrophil extracellular traps (NETs) contribute to the pathogenesis of many autoimmune diseases, including vasculitis. Though neutrophils, and NETs, can break self-tolerance by being a source of autoantigens for autoantibodies in anti-neutrophil cytoplasmic antibody (ANCA)-associated vasculitis, playing a key role in driving the autoimmune response, the role of neutrophils and NETs in large vessel vasculitis, including giant cell arteritis (GCA), is not well understood. In this review, we summarize the current insight into molecular mechanisms contributing to neutrophil-mediated pathology in small and medium vessel vasculitis, as well as provide potential translational perspectives on how neutrophils, and NETs, may partake in large vessel vasculitis, a rare disease entity of unclear pathogenesis.

## Introduction

Neutrophils are important mediators of host defense against pathogens including bacteria, fungi, and protozoa, and one of the major arms of the innate immune system (1). Recruitment and activation of neutrophils at sites of tissue infection leads to killing of the pathogen through several cytotoxic mechanisms including phagocytosis, production of reactive oxygen species (ROS) and release of neutrophil extracellular traps (NETs) (2). NETs are extruded webs of decondensed nuclear DNA, citrullinated histones and granule proteins (3) including neutrophil elastase (NE), calprotectin (also known as S100A8/A9) and myeloperoxidase (MPO) (4) able to trap and eliminate pathogens.

Other than pathogenic triggers, several host-derived components, including inflammatory cytokines and chemokines, IgG immune complexes (ICs), monosodium urate crystals, and cholesterol crystals, as well as activated platelets, have been shown to induce NET formation *in vitro* and *in vivo* (5–7). The IgG ICs induce NET release after binding to FcγRIIA on neutrophils (8, 9). The capacity of ICs and inflammatory cytokines to induce NET formation is consistent with excessive NET formation being observed in several autoimmune and inflammatory conditions, including gout, rheumatoid arthritis (RA), psoriasis, systemic lupus erythematosus (SLE), juvenile dermatomyositis (JDM), and anti-neutrophil cytoplasmic antibody (ANCA)-associated vasculitis (AAV) with levels of NETs often linked to disease activity and severity (10–13).

However, little is known about the significance of neutrophils in the pathogenesis of other types of vasculitis such as Takayasu’s arteritis (TAK) and giant cell arteritis (GCA). The objective of this review is to shed light into the role of neutrophils in the pathogenesis of systemic vasculitides, with a specific focus on the potential diverse vasculopathic and immunogenic effects of neutrophils in the development of small, medium, and large sized vessel vasculitides.

## Role of Neutrophils in Small Vessel Vasculitis

### Neutrophils and Anti-Neutrophil Cytoplasmic Antibody-Associated Vasculitis

AAV is a group of small vessel vasculitis characterized by small blood vessel inflammation and presence of circulating ANCAs. There are three subtypes of AAV that have been described so far, namely granulomatosis with polyangiitis (GPA), microscopic polyangiitis (MPA), and eosinophilic granulomatosis with polyangiitis (EGPA) (14). Constitutional symptoms such as malaise, fever, and weight loss are common to all three subtypes of AAV. GPA is characterized by the presence of necrotizing granulomatous inflammation most commonly in the upper or lower respiratory tract, but can also occur in other organs such as skin, orbit or the meninges (15). Kidney involvement predicts higher mortality and morbidity (16).

MPA is often characterized by rapidly progressive glomerulonephritis (RPGN). The most characteristic pulmonary involvement in MPA is hemorrhagic alveolar capillaritis, which histologically is characterized by focal areas of neutrophils in alveolar capillaries and lysis of capillaries with leukocytoclastic debris (17). EGPA is characterized by asthma, eosinophilia, nasal polyps, and eosinophilic pulmonary infiltrates. Other organs that are affected include peripheral and central nervous system, skin, gut, and heart. Renal involvement is usually associated with positive ANCA (18). What differentiates MPA from GPA and EGPA is the absence of necrotizing granulomatous inflammation of the respiratory tract (19). Acute lesions of GPA are characterized by neutrophilic infiltrates forming micro-abscesses and presence of multinucleated giant cells with focal accumulations of fibrinoid material. Acute lesions of MPA are characterized by leukocytoclasia, and vessel wall necrosis with accumulation of fibrin following activation of coagulation factors. As the lesions progress there is accumulation of monocytes, macrophages, and T lymphocytes and transformation to more fibrotic lesions. The acute vasculitic phase of EGPA is characterized by a much more intense eosinophilic infiltration of the necrotizing granulomatous inflammation that resembles that of GPA (17). AAV are designated pauci-immune vasculitides as immunohistology shows few or no immunoglobulin and C3 deposits at the inflammatory lesions (20).

The targets of the ANCAs in AAV are primarily myeloperoxidase (MPO) and proteinase 3 (PR3), granular enzymes within the neutrophils. The association of the three subtypes of AAV with the type of ANCA varies. Patients with GPA are more likely to have antibodies to PR3 (21). A defect of the gene for a1-antitrypsin (SERPINA) and/or inherited predisposition for an increased expression of the PRTN3 gene may trigger the synthesis of anti-PR3 ANCA that bind to the surface of neutrophils in GPA (22). PRTN3 and MPO genes in neutrophils of AAV patients have a distinct pattern of histone modifications, implicating epigenetic mechanisms in the expression of those autoantigen genes (23). The majority of patients with MPA are positive for MPO-ANCA. Approximately 45% of patients with EGPA test positive for MPO-ANCA correlating with renal involvement (21). Lactoferrin is another antigen of ANCAs and patients with EGPA who had positive anti-lactoferrin antibodies had significantly higher frequency of renal involvement, serum CRP levels, and Birmingham Vasculitis Activity Score (BVAS) (24). Patients with EGPA showed enhanced ability to produce NETs compared to healthy subjects with no regard to the ANCA status (25).

**Figure 1:**
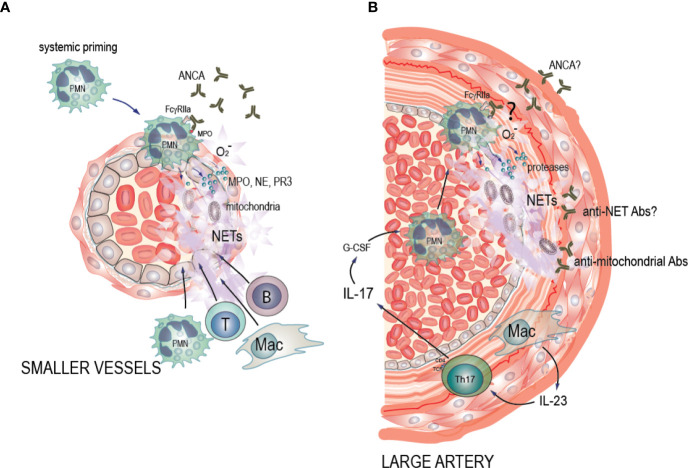
Potential pathogenic mechanisms of NET formation in ANCA-associated vasculitis (AAV) and large vessel vasculitis (LVV). **(A)** AAV: Pro-inflammatory stimuli such as TNF-α, LPS and C5a cause neutrophil priming, with increased expression of the antigens MPO, PR3, and other neutrophil granule proteins to the cell surface where ANCA can bind to them. Soluble and cell-bound immune complexes of ANCA and ANCA antigens then bind and crosslink FcgRIIA on the neutrophil surface, activating the oxidative burst machinery and driving degranulation of MPO, NE, and PR3, decondensation and extrusion of nucleosomal chromatin from the cells leading to NET formation and eventually neutrophil death. This in turn drives a necrotizing inflammation that results in endothelial cell death, vascular leakage, fibrin deposition, and a subsequent monocyte and macrophage recruitment. This phase eventually evolves into a fibrin and collagen-rich lesion, which may resolve if the initial inflammation was limited, or become permanent scar tissue with lingering chronic mononuclear cell infiltrates with B and T cells in ectopic germinal center-like structures. In these instances, the inflamed artery may be permanently occluded. **(B)** LVV: We hypothesize that IL-23 that is excreted by the macrophages in the vessel wall induces Th17 cells. Th17 cells then produce IL-17 that up-regulates G-CSF leading to neutrophil recruitment in circulation, and activation of neutrophils. We hypothesize that the presence of ANCA antibodies of unknown entity, may induce neutrophil activation. Alternatively, ANCA antibodies may bind to NETs and form anti-NET antibodies preventing NET degradation as well as creating neutrophil-activating immune complexes. Activated neutrophils then adhere to the endothelium of the affected arteries. Subsequently, neutrophils interact with the damaged endothelium and undergo cell death characterized by NET formation. Endothelial damage perpetuates neutrophil activation *via* alternative complement activation in a vicious circle. Additionally, we hypothesize that release of mitochondrial components during NET formation leads to formation of anti-mitochondrial antibodies, contributing to vessel wall damage and inflammation.

A prerequisite for binding of the autoantibodies to their target molecules is the exposure of the antigens. This could occur either upon up-regulation of the antigens on the cell surface and/or upon cell death and release of the antigens in the extracellular environment, such as during NET formation (26). ANCA activates neutrophils to degranulate (27), produce ROS (28) and extrude chromosomal DNA in the form of NETs (29, 30). Neutrophil activation by ANCA depends on their ‘priming’ by cytokines like tumor necrosis factor-alpha (TNF)-α, lipopolysaccharide (LPS) (31, 32), or complement factor 5a (C5a) (33, 34). These stimuli not only induce expression of endothelial selectins that enable interaction with neutrophils resulting in their rolling, intravascular crawling, and transcellular migration (35), but also result in increased cell surface expression of MPO, PR3, and other neutrophil granule proteins to the neutrophil cell surface where ANCA can bind to them (36). Upon binding of ANCAs to the antigens, the Fc part of the autoantibody will engage FcγRs, resulting in neutrophil activation, promoting firm neutrophil adhesion to the endothelium, NET formation, and inflammatory damage to the endothelium (**Figure 1A**). Endothelial damage perpetuates neutrophil recruitment and activation *via* alternative complement activation in a vicious circle (17, 35, 37). High serum levels of complement split products C3a and C5a have been found in patients with active AAV (34, 38). Avacopan, a C5a receptor (C5aR) antagonist, prevented MPO-ANCA-induced glomerulonephritis (GN) in a murine model of AAV (39). Further, C5aR inhibition with avacopan effectively replaced high-dose glucocorticoids in AAV (40).

Another mechanism of autoantigen exposure is release of MPO and PR3 from activated neutrophils at inflammatory sites. The released antigens bind to endothelial cells, resulting in subsequent *in situ* formation of immune complexes (2, 17). Neutrophils, interacting with the activated/damaged endothelium, will induce leukocytoclasia, as well as undergo cell death characterized by formation of NETs (41). NETs are also likely to be involved in the loss of T and B cell tolerance to both MPO and PR3 by activating dendritic cells (DCs). When myeloid DCs were loaded with NET components and injected into naïve mice they were able to induce ANCA and autoimmunity (42). Similar findings are seen also in other diseases, including rheumatoid arthritis, where NET-derived citrullinated epitopes can be presented by fibroblast-like synoviocytes and promote activation of antigen-specific CD4+ T cells (43). NETs are not only present in organ lesions from patients with AAV, but are also found in their circulation (44). The elevated levels of NETs in the circulation of AAV patients could be attributed to reduced clearance of NETs (45).

However, excessive serum-mediated *ex vivo* NET formation has also been reported in PR3-ANCA and MPO-ANCA positive AAV patients (46). Thus, both reduced clearance and excessive NET formation may contribute to the elevated levels of circulating NETs observed in these patients. The exact identity of the NET-inducing agent(s) in AAV is not known. Though prior work have implicated MPO ANCA IgG in NET induction (45), other studies found that NETs were induced in an ANCA-independent process (46). These findings suggest that the presence of serum factors other than ANCA IgG might preclude NET degradation and/or enhance NET formation in both PR3- and MPO-ANCA-associated AAV. In another study that compared mechanisms of NET formation between AAV and SLE, it was also shown that AAV-induced NET formation occurred independently of IgG ANCAs whereas SLE ICs induced NET formation *via* Fcγ receptor signaling pathways (47). Further, the presence of inhibitory antibodies, including anti-DNA antibodies can likely contribute to a low ability for NET degradation in MPO-ANCA-associated MPA serum, similar to what has been described in SLE (48). Impaired NET degradation in active SLE due to presence of DNase-I inhibitor and anti-NET antibodies leads to increased NET levels that are related with disease activity and kidney damage (49). Indeed, patients with active SLE who had an impaired ability to degrade NETs had more anti-NET antibodies that further increased C1q deposition (48).

NETs are prominent inducers of inflammation, including through release of mitochondrial components, signaling through DNA sensing TLR9 as well as the cGAS-STING pathway (50). Neutrophil activation in SLE results in release of oxidized mitochondrial DNA, driving type I interferon production (51). Other mitochondrial components, such as cardiolipin, that are released by NETs could be another important source of circulating autoantigens in both AAV and SLE (52). Those NET-derived autoantigens may act to further amplify the inflammatory process through formation of local immune complexes. Presence of anti-cardiolipin antibodies was reported in 40% and 57% of patients with GPA and MPA, respectively, but did not correlate with the presence of ANCA in any of those disease groups (53).

Another prominent neutrophil activation marker, calprotectin (also known as MRP8/14 and S100A8/A9), is elevated on the neutrophil cell surface in acute AAV as compared to healthy controls (54, 55). Further, even in remission, AAV patients have elevated levels of serum calprotectin, implying subclinical disease activity triggered by neutrophil activation. Similarly to levels of NETs, serum levels of calprotectin did not correlate with ANCA level (54). However, it should be noted that coagulation leads to release of calprotectin from neutrophils, as well as from platelets (11, 56). As such, serum levels of calprotectin are in large artificial and do not represent the true levels of circulating calprotectin in the patients. Further studies, assessing plasma levels of calprotectin (and NETs) are warranted to determine the association between ANCA levels and neutrophil activation.

Expression of calprotectin has also been observed in the kidneys of patients with AAV-associated GN, indicating local neutrophil activation (57). Other neutrophil-associated markers, including NE, a neutrophil gene signature, and presence of low-density granulocytes (LDGs) are all associated with disease activity in AAV (58, 59). LDGs in AAV are heterogeneous, displaying both mature and immature granulocytes and were refractory to MPO-ANCA-induced oxidative burst despite expressing MPO, indicating alternative roles for LDGs in AAV pathogenesis (60, 61). Inflammatory components, including calprotectin, are known to promote neutrophil survival (62), with neutrophils having prolonged lifespan within the inflammatory lesions as demonstrated in a human transgenic PR3 mouse model (20). The signaling pathways *via* which calprotectin is acting, are primarily TLR4 and RAGE, resulting in induction of pro-inflammatory cytokines such as TNF-α, interleukin (IL)-1β, IL-6, IL-8, and IL-23, chemokines, and adhesion molecules amplifying the inflammatory response and leading to leukocyte adhesion to the endothelium (63).

Defective clearance of apoptotic neutrophils within necrotizing granulomatous inflammation of GPA, results in their necrosis with the release of pro-inflammatory cytokines, and damage-associated molecular patterns (DAMPs) (64). DAMPs such as high-mobility-group-protein B1 (HMGB1) and the auto-antigen PR3 are expressed on the surface of apoptotic neutrophils contributing to immunogenic responses (65). HMGB1 participates in ANCA-induced NET formation through interaction with Toll like receptor (TLR)2, TLR4 and the receptor for advanced glycation end products (RAGE) and the process is NADPH oxidase dependent (66). Other neutrophil- and NET-associated molecules and mediators in AAV are listed in **Table 1** (76, 77, 79, 80).

**Table 1:** Neutrophils mediators and/or activators in AAV and other vasculitides.

NET-mediators	Neutrophil localization	Type of vasculitis	Role in vasculitis	Ref.
MPO	Azurophilic granules	AAV	Autoantigen in	(17, 26, 29, 30, 74)
		CLAAS	AAV and CLAAS.	
			PTU- MPO AAV		Injection of PTU-
					NETs causes AAV in rats.	(77)
					KD		Elevated levels in KD.	(94)
					DADA2		Up-regulated MPO expression in DADA2.	(91)
					BD		Participation in formation of DNA complexes in BD.	(122)
PR3	Azurophilic granules				AAV		Autoantigen in AAV and CLAAS.	(17, 26, 29, 30, 74)
					CLAAS			
NE	Azurophilic granules				AAV		Anti-elastase antibodies are present in AAV.	(59, 60)
					CLAAS		Target of ANCAs in CLAAS.	(74, 75)
					KD		Elevated levels in KD.	(94)
Calprotectin	Cytosolic protein content of neutrophils				AAV		Promotion of neutrophil survival in AAV.	(63)
							Associaed with proliferative GN.	(59)
TNF-α	–				AAV		‘Priming’ of neutrophils and enhanced transcription of the TNF-a gene in peripheral blood mononuclear cells from patients with AAV.	(32, 33)
					LVV		Possible recruitment, activation and survival of neutrophils in LVV.	(112)
LPS	–				AAV		‘Priming’ of neutrophils in AAV.	(32)
C5a	–				AAV		‘Priming’ of neutrophils for ANCA induced NETs.	(34, 36)
					LVV		Possible interaction with its cellular receptor on surface of neutrophils after priming by G-SCF leading to NETs in LVV.	(113)
DNase I inhibitor	–				AAV		Possible low NET degradation in MPO AAV.	(48–50)
					PTU- MPO AAV		Treatment of neutrophils with PTU led to NET formation resistant to DNase I.	(75)
Anti-NET antibodies	–				AAV		Possible impaired NET degradation in AAV	(47–49)
Cardiolipin	Mitochondria				AAV LVV		Possible circulating autoantigen in AAV and LVV.	(53, 54, 102)
HMGB1	Nucleus				AAV		Interaction with the receptors TLR2, TLR4 and RAGE when expressed on the surface of apoptotic neutrophils in AAV.	(66, 67)
Azurocidin	Azurophilic granules				AAV		Autoantibodies present in AAV.	(68)
Cathepsin G	Azurophilic granules				AAV		Autoantibodies present in AAV.	(69)
Lactoferrin	Secondary granules				AAV		Atypical ANCA in AAV.	(25)
					IgAV		Binding of IgA ICs to neutrophils leads to release of lactoferrin and NETs in IgAV.	(82)
TF	Acidified autophagosomes				AAV		Induction of thrombosis and inflammation in AAV.	(70)
Cathelcidin LL37	Nuclear				AAV		Increased levels in AAV patients particularly those with crescentic formation	(71)
Anti-LAMP-2	Lysosomal membrane of granules				AAV		Atypical ANCA in AAV	(69)
Lysozym C	Secondary granules				AAV		Atypical ANCA in AAV	(69)
A1, A3 ARs	G-protein coupled receptors				DADA2		Regulation of neutrophil function by promoting neutrophil chemotaxis towards inflammatory stimuli.	(84, 85, 92)
							Increased NET formation by binding of adenosine to A1 and A3 ARs.	
IL-1β, IL-8	–				LVV		Treatment of neutrophils from healthy subjects with IL-1β, or IL-8 enhanced free radicals generation and NETs formation in LVV	(111)
IL-6, IL-17	–				LVV		Neutrophil activation in LVV	(102)
GCSF	–				LVV		‘Possible priming’ of neutrophils in LVV.	(116, 117)
					Drug induced LVV			

MPO, myeloperoxidase; PR3, proteinase 3; AAV, anti-neutrophil cytoplasmic antibody (ANCA)-associated vasculitis; CLAAS, cocaine/levamisole-associated autoimmunity syndrome; PTU-MPO AAV, prophylthiouracil-MPO AAV; PTU-NETs, propylthiouracil-neutrophil extracellular traps; KD, Kawasaki disease; DADA2, deficiency of adenosine deaminase type 2; BD, Behcet’s disease; NE, neutrophil elastase; GN, glomerulonephritis; TNF-α, tumor necrosis factor-alpha; LVV, large vessel vasculitis; LPS, lipopolysaccharide; C5a, complement factor 5a; anti-NET antibodies, anti-neutrophil extracellular traps antibodies; HMGB1, high-mobility-group-protein B1; TLR2, Toll like receptor 2; TLR4, Toll like receptor 4; RAGE, receptor for advanced glycation end products; IgAV, IgA vasculitis; TF, Tissue factor; Anti-LAMP-2, anti lysosomal membrane protein 2; ARs, adenosine receptors; IL-1β, interleukin -1-beta; IL-8, interleukin-8; IL-6, interleukin-6; IL-17, interleukin-17; GCSF, granulocyte colony stimulating factor.

### Neutrophils and Drug-Induced Anti-Neutrophil Cytoplasmic Antibody-Associated Vasculitis

**Figure 2:**
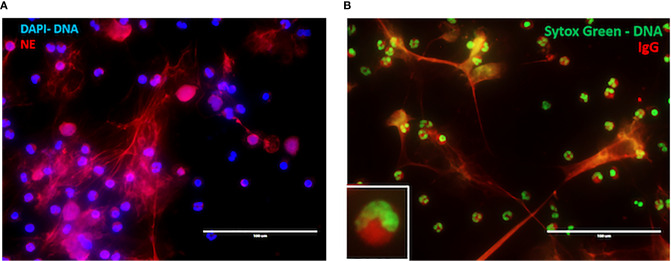
NET formation in CLAAs. **(A)** Immunofluorescence microscopy (IF) illustrating levamisole-induced NET formation. Staining was for DNA (blue) and neutrophil elastase (NE, red). **(B)** IF demonstrating CLAAS IgG binding to cytosolic components, as well as NET-derived antigens. Staining was for DNA (green) and CLAAS IgG (red). Those are alternative images prepared according to the methods outlined in ref (72).

There are several studies that provide a tantalizing link between NET formation and drug-induced vasculitis. Cocaine and levamisole have independently been associated with the development of ANCAs (87), exerting toxic effects on active illicit cocaine users such as vasculitic purpura and neutropenia (88). Patients with cocaine/levamisole-associated autoimmunity syndrome (CLAAS) show different patterns of ANCA, including enriched presence of anti-MPO/anti-PR3 dual reactivity. Notably the presence of c-ANCA pattern in that patient population was associated with increased mortality (88). In CLAAS, a dominant target of ANCAs is NE (**Table 1**). Cocaine and levamisole can induce formation of NETs enriched in NE [**Figures 2A, B**, alternate images prepared as in (72)] and, potentially, inflammatory mitochondrial DNA (72). It was demonstrated recently, that in levamisole-induced autoimmunity NET formation is triggered by levamisole through engagement of M3 muscarinic receptors on neutrophils (89).

Prophylthiouracil (PTU) is a drug that commonly induces anti-MPO seropositivity and AAV in humans (68). It was recently demonstrated that PTU induced MPO-ANCA IgG antibodies, as well as primed neutrophils to undergo NET formation (90). Neutrophils that were treated with PTU developed an abnormal, globular conformation during NET formation in that they were relatively resistant to DNase I digestion. Furthermore, injection of these PTU NETs into rats led not only to ANCA production (**Table 1**), but also pulmonary capillaritis and GN reminiscent of human vasculitis (91).

Drug-induced AAV has been reported in association with hydralazine (87). Hydralazine was able to trigger NET formation by modulating calcium release from intracellular stores, implying the role of NETs in the pathogenesis of drug-induced autoimmunity. NET formation induced by hydralazine did not interfere with NET degradation and required peptidylarginine deiminase 4 (PAD4) activation (92).

#### Neutrophils and IgA Vasculitis

IgA vasculitis (IgAV) is also referred to as Henoch-Schönlein purpura, and is characterized by immunoglobulin A1 (IgA1)- immune deposits in the small vessels of the skin, gastrointestinal tract, and kidneys. Manifestations of the disease include palpable purpura or petechiae, polyarthralgias, abdominal pain, and glomerulonephritis (93). The binding of IgA immune complexes to FcαRI (CD89) on neutrophils results in phagocytosis, production of ROS, release of granules that contain lactoferrin, and release of NETs (78). NETs were found around inflamed vessels in IgA vasculitis (**Table 1**). Their presence was highest after the onset of vasculitis but decreased progressively with disease course. There was a strong correlation of NETs with the production of ROS (94).

## Role of Neutrophils in Medium Vessel Vasculitis

### Neutrophils and Deficiency of Adenosine Deaminase Type 2

Human neutrophils express A1, A2, and A3 adenosine receptors (ARs) and can release adenosine at inflammatory sites. Adenosine-mediated stimulation of A1 and A3 ARs on neutrophils can regulate neutrophil function by promoting neutrophil chemotaxis towards inflammatory stimuli (**Table 1**) and promote phagocytosis (81, 82). On the other hand, A2 receptors inhibit respiratory burst that is considered a critical, early step in NET formation (95) and inhibit neutrophil function (96).

The adenosine deaminase type 2 (*ADA2*) gene encodes a dimeric protein that after its secretion into the extracellular space functions as a deaminase to convert adenosine to inosine and 2′-deoxyadenosine to 2′-deoxyinosine (97). DADA2 is a monogenic vasculitis syndrome that is caused by autosomal-recessive loss-of-function mutations in the *ADA2* gene, previously known as *cat eye syndrome chromosome region*, *candidate* 1(*CECR1*). DADA2 manifests with fevers, polyarteritis nodosa (PAN), livedo racemosa, elevation of acute phase reactants, early-onset of ischemic or hemorrhagic strokes, and mild immunodeficiency (98, 99). Patients with DADA2 have not only similar clinical but also histo-pathologic features (non-granulomatous, necrotizing arteritis of small-or medium sized muscular arteries) of systemic PAN (99).

Up-regulated neutrophil signature on genome-wide microarray analysis has been observed in the peripheral blood of patients with DADA2. Interestingly, circulating neutrophils showed increased expression of MPO (**Table 1**), leading to the speculation that ADA2 may prevent MPO expression (70). It was recently reported that NET formation is enhanced in DADA2, and macrophage secretion of ADA2 is a significant regulator of adenosine mediated NET formation. Lack of ADA2 activity in patients with DADA2 leads to accumulation of extracellular adenosine and subsequent triggering of NET formation, particularly in neutrophils from female patients, by binding to A_1_ and A_3_ ARs and through NADPH oxidase- and PAD-dependent pathways (83).

### Neutrophils and Kawasaki Disease

KD is a multi-systemic vasculitis that mainly affects the medium and small vessel arteries, but aorta and large arteries may also be affected. It is characterized by fever accompanied by lymphadenopathy, rash, conjunctivitis, and oropharyngeal mucosal changes (100). In the acute phase of KD, plasma levels of NE and MPO are increased (**Table 1**), suggesting that neutrophil activation may contribute to the immunopathogenesis of KD vasculitis (69). Of note, neutrophils from KD patients undergo spontaneous *ex vivo* NET formation upon isolation, similar to what has been observed in SLE. These findings suggest that circulating neutrophils may be primed by pro-inflammatory mediators to undergo NET formation in KD vasculitis (101). So far, there are no reports on levels of circulating NETs in KD.

## Role of Neutrophils in Large Vessel Vasculitis

### Neutrophils in TAK and Giant Cell Arteritis

TAK and GCA are the two major forms of LVV characterized by vascular inflammation and resultant damage of the aorta and branch arteries (102). Clinical manifestations of LVV include headache, lightheadedness, carotidynia, vision loss, stroke, transient ischemic attack (TIA), syncope, and upper limb claudication (103, 104). As demonstrated in a recent study, the most common symptom in TAK patients was arm claudication (52%) whereas in patients with GCA it was blurred vision (37%) (105).

Neutrophils play an essential role in the pathogenesis of LVV. Local recruitment and infiltration of neutrophils have been seen in histological specimens of aorta from patients with TAK, as well as adventitia and media of affected arteries in GCA, contributing to local inflammation and disease progression (75, 106–108). Increased levels of neutrophils was observed in the circulation of TAK patients that was positively correlated with disease activity (109).

Though negative to defined antigens, i.e. proteinase-3, human leucocyte elastase, myeloperoxidase, and lactoferrin as detected by ELISA, GCA patients have strong reactivity to neutrophil cytoplasmic antigen(s) of unknown identity (110). Anti-mitochondrial antibodies, and specifically anti-cardiolipin antibodies of the IgG subtype (**Table 1**), have also been reported in 51.5% of GCA patients at disease onset (111). It is so far unclear whether those unknown neutrophil cytoplasmic and mitochondrial antigens may form immune complexes and induce NET formation *via* Fcγ receptor-mediated mechanisms.

Potential involvement of neutrophils in GCA pathogenesis and relapse was suggested when at week 24 after glucocorticoid therapy, GCA neutrophils were unable to suppress T-cell responses, implying re-emergence of vascular inflammation. Reduction in T-cell suppressor neutrophils was reproduced *in vitro*, after using concentrations of IL-6 and IL-17 equivalent to those measured in GCA plasma samples. This reduction correlated with attenuated inhibition of lymphocyte proliferation (75).

IL-17-producing Th17 T cells are markedly increased in GCA but sensitive to glucocorticoid-mediated suppression (112). IL-17 inhibition with secukinumab may be an option as maintenance therapy for glucocorticoid-free remission in GCA (113). It was recently demonstrated that immature neutrophils from GCA patients amplified vascular damage *via* production of high levels of extracellular reactive oxygen species leading to enhanced permeability of endothelial barrier in an *in vitro* neutrophil- endothelial co-culture system (114).

IL-1β is highly expressed in the inflamed arterial walls of patients with GCA (115). NET production can be induced by IL-1β *in vitro* (84). Pro-inflammatory cytokines IL-17, IL-8, interferon γ, and TNF-α also play major roles in the recruitment, activation and survival of neutrophils in inflammation (73), and those cytokines were significantly increased in TAK (107) (**Table 1**). Treatment of neutrophils of healthy objects with TNF-α, IL-1β, or IL-8, results in production of free radicals and NET formation by activation of NADPH oxidase (84). This finding emphasizes the significance of those cytokines in the potential release of NETs in systemic inflammatory response syndromes like LVV (**Figure 1B**).

### Neutrophils in Drug-Induced Large Vessel Vasculitis

Granulocyte colony stimulating factor (G-CSF) may rarely cause LVV (74, 116, 117). G-CSF is a myeloid growth factor that can be produced by monocytes, macrophages, fibroblasts, and endothelial cells. One of the possible mechanisms by which exogenous administration of G-CSF may induce LVV includes stimulation of the proliferation and differentiation of neutrophil precursors and enhancement of neutrophil chemotaxis (85). G-CSF may have a priming effect in human neutrophils (**Table 1**). Interestingly, viable human neutrophils after priming with granulocyte/macrophage colony-stimulating factor (GM-CSF) and subsequent stimulation of TLR4 or C5a receptor were able to generate NETs (86). C5a *via* interaction with its cellular receptor on neutrophil surface leads to changes in the neutrophil cell shape and membrane formability that allows the neutrophil not only to transform into a migratory cell and invade inflammatory sites but also clear pathogens and debris (118). A randomized double blind placebo-controlled phase 2 trial is currently investigating mavrilimumab that is a fully humanized monoclonal antibody targeting GM-CSF receptor alpha (GM-CSFRα) (119) in giant cell arteritis.

## Role of Neutrophils in Variable Vessel Vasculitis

### Neutrophils and Behcet’s Disease

BD is a chronic systemic vasculitis manifested by a triad of relapsing iritis, aphthous stomatitis and genital ulcers (120). It can affect other organs such as skin, mucous membranes, gastrointestinal tract, joints, the central nervous system and blood vessels, with a neutrophil-dominating infiltration around vasa vasorum being very characteristic of vasculo-BD (121). In a recent study, patients who had active BD and vascular involvement had higher levels of cell free DNA (cfDNA) and MPO-DNA complexes in their serum compared to patients with inactive BD and no vascular involvement (**Table 1**). Notably, purified neutrophils from patients with BD underwent spontaneous NET formation compared to healthy donors (HD). This is likely clinically significant as markers of NET formation was associated with thrombin generation in BD. Further, NETs were present in areas of vasculitic inflammation and thrombosis (71).

## Conclusions

This review unravels the role of neutrophils in the pathogenesis of systemic vasculitides. Without doubt, neutrophils are considered dominant players in the pathophysiology of systemic autoimmune diseases. Although the role of neutrophils in small vessel vasculitis has been fairly well established, there is an unmet need of defining the molecular signaling pathways and mechanisms promoting neutrophil-mediated inflammation and damage in both small vessel vasculitis as well as LVV. Elucidating the effect of neutrophils on those distinct disorders and the pathogenic mechanisms by which NETs are generated will not only enhance our knowledge about the immunopathogenesis of those complex diseases but may also lead to discovery of novel diagnostic and prognostic biomarkers, as well as targets for pharmaceutical interventions in the future.

## Author Contributions

DM, TM, and CL substantially contributed to this review with regard to content and structure of the manuscript. All authors contributed to the article and approved the submitted version.

## Funding

Our work is supported by Pfizer US Pharmaceuticals Group grant with sponsor award number 53857367 (DM), NIH grants R21AR075134 (TM), R01 AR074939 (TM), and R21 AR077266 (TM), and NIH grants 1R21EY029391 (CL) and R21AR075129 (CL). The funder bodies were not involved in the study design, collection, analysis, interpretation of data, the writing of this article or the decision to submit it for publication.

## Conflict of Interest

TM has received consulting fees from Cugene, Kiniksa, Miro Bio, and QiLu Pharmaceuticals, has an ownership share in Amdax, and has received research funding from Gilead Sciences. CL has received research funding from Exagen Inc and Eli Lilly.
